# Discussing prognosis with older people with musculoskeletal pain: a cross-sectional study in general practice

**DOI:** 10.1186/1471-2296-10-50

**Published:** 2009-07-07

**Authors:** Christian David Mallen, George Peat

**Affiliations:** 1 Arthritis Research Campaign National Primary Care Centre, Keele University, Keele, Staffordshire, ST5 5BG, UK

## Abstract

**Background:**

Prognosis has been described as an important but neglected branch of clinical science. While patients' views have been sought in the context of life-threatening illness, similar research is lacking for patients presenting with common, non-life-threatening musculoskeletal complaints. The aim of this study was to gauge whether and why older patients with musculoskeletal pain think prognostic information is important, and how often they felt prognosis was discussed in the general practice consultation.

**Methods:**

A cross-sectional survey of consecutive patients aged 50 years of over presenting with non-inflammatory musculoskeletal pain to 5 Central Cheshire general practices. The frequency of responses to the prognostic questions were described and the association with sociodemographic, presenting pain complaint, and psychosocial variables explored using logistic regression.

**Results:**

502 participants (77%) responded to the postal questionnaire. 165 (33%) participants reported discussing prognosis in the consultation with their GP. Discussions about prognosis were more often reported by male patients (OR 1.72, 95% CI 1.09, 2.71) and those for whom this was their first consultation (OR 1.81, 95% CI 1.16, 2.80). 402 (82%) participants thought that prognostic information was important. This was highest among those currently in paid employment (OR 2.95, 95% CI 1.33, 6.57). The reasons patients gave for believing prognostic information was important included 'knowing for the sake of knowing' and planning future activity. Reasons for not believing prognostic information to be important included the belief that progression of pain was inevitable and that nothing could be done to help.

**Conclusion:**

Prognostic information is thought to be important amongst older people with musculoskeletal pain yet discussions occur infrequently in primary care. Barriers to effective prognostic communication and the exact information needs of patients are still unknown and warrant further research.

## Background

Prognosis is the forecast of the probable course and (or) outcome of a disease and has been described as an important but neglected branch of clinical science [1-3]. Prognostic models and prediction rules for several musculoskeletal conditions are now beginning to emerge but how many patients actually want to be given information on the likely outcome of their condition? While patients' views have been sought in the context of life-threatening illness [4-7], similar research is lacking for patients presenting with common, non-life-threatening musculoskeletal complaints.

We present the findings of a survey of older adults presenting to the general practitioner (GP) with musculoskeletal pain. Our objectives were to determine what proportion of patients think prognostic information is important, the reasons they give for (not) why they feel prognostic information is important, and to ascertain how often patients feel that their prognosis is discussed in the consultation.

## Methods

### Design and setting

We conducted a survey of consecutive patients aged 50 years and over consulting one of five Central Cheshire general practices with non-inflammatory musculoskeletal pain between September 2006 and March 2007. Data for this analysis is restricted to the cross-sectional baseline questionnaire data, although the PROG-RES study is a longitudinal cohort, with multiple data collection points (GP consultation, baseline questionnaire, three, six, twelve months) [8]. Ethical permission was obtained from the Central Cheshire Local Research Ethics Committee (REC Reference: 06/Q1503/60). Full details of the study design have been previously published [8].

Patients consulting with eligible Read codes were identified through weekly electronic downloads from the general practice records. Read codes are a hierarchy of morbidity, symptom and process codes that enable UK primary care workers to code consultations by either presenting symptom, the working or established diagnosis or the procedure undertaken [9].

Potential participants were mailed a 16-page self-completion questionnaire within one week of consulting their doctor. Those not responding to the initial questionnaire were sent a reminder postcard at two weeks and a repeat questionnaire at four weeks.

### Questionnaire content

Full details of the questionnaire content have been presented elsewhere [8]. In brief, the questionnaire included questions on demographics (date of birth, gender, occupation, level of education), pain (pain manikin [10], Chronic Pain Grade [11], mode of onset [12], episode duration [13]), psychological factors (coping strategies [14], anxiety and depression [15], depression screening questions [16]), social characteristics (living alone, social support [17]) and general health [18].

### Outcome measures

The following questions about the provision of, and desire for, prognostic information were asked in the questionnaire:

• 'Did your doctor tell you what was likely to happen to this pain?' (Response options: Yes/No/Don't know)

• 'Do you think it is important to know what is likely to happen to this pain?' (Response options: Yes/No/Don't Know)

• If you answered yes (no) to the question above, please explain in your words why you think it is important (not important) to know what is going to happen to your pain (Response options: free text).

For the analysis described below, 'No' and 'Don't Know' categories were combined.

### Statistical analysis

Responses to the first two prognostic questions were summarised as simple frequencies Binary logistic regression was used to investigate the role of sociodemongraphic status, characteristics of the presenting pain complaint, and psychosocial variables as determinants of recalled prognostic discussion and perceived importance. Results were expressed as odds ratios (OR) with 95% confidence intervals (95% CI), both crude and adjusted for all other covariates (complete case analysis).

Content analysis was used to identify themes in the free text responses provided by participants to the question asking about the importance of knowing what is likely to happen to their pain over the next six months. Content analysis has been broadly defined as "a research technique for the objective, systematic and quantitative description of the manifest content of communication" [19]. Content analysis can be applied to any written or recorded communication. The content of the free text responses were transcribed, read by the lead author and grouped into themes on the basis of key words.

## Results

A total of 502 (77%) of 650 potential participants identified from the electronic record download responded to the self-completion questionnaire (mean age 65 years, range 50–97; 61% female). The most common presenting complaints were back pain (29%), shoulder pain (26%) and knee pain (25%). 32% (n = 155) of participants described their physical health as being poor or fair, 14% (n = 67) had moderate or severe depressive symptoms and 21% (n = 103) having moderate or severe anxiety symptoms.

165 (33%) participants recalled discussing prognosis in the consultation with their GP (299 recalled no such discussion, 31 'don't know', 7 missing). The proportion recalling prognostic discussion was similar across all participating practices. Discussions about prognosis were more often reported by male patients (adjusted OR 1.72; 95% CI 1.09, 2.71) and those for whom this was their first consultation (1.81; 1.16, 2.80) (see Table 1). There was no association with patient age, living arrangement, employment status, general health status, pain severity, anxiety, depression, or self-reported social support.

**Table 1:** Determinants of (patient-recalled) prognostic discussion in the consultation

	Prognostic discussion recalled N (%)	Crude OR (95% CI)	Adjusted† OR (95% CI)
Age (years)			
50–59	55 (32)	1	1
60–69	60 (36)	1.19 (0.76, 1.87)	1.04 (0.59, 1.81)
70–79	35 (33)	1.03 (0.62, 1.73)	1.07 (0.53, 2.12)
80+	15 (31)	0.94 (0.47, 1.87)	0.95 (0.37, 2.44)
Gender			
Female	87 (29)	1	1
Male	78 (41)	1.70 (1.16, 2.49)	1.72 (1.09, 2.71)
First consultation			
No	84 (28)	1	1
Yes	80 (42)	1.84 (1.25, 2.69)	1.81 (1.16, 2.80)
Self-rated general health			
Excellent/very good/good	119 (36)	1	1
Fair/poor	44 (29)	0.73 (0.48, 1.11)	0.76 (0.46, 1.27)
Chronic Pain Grade			
I	29 (45)	1	1
II	30 (31)	0.55 (0.29, 1.05)	0.64 (0.32, 1.27)
III	32 (30)	0.52 (0.25, 0.99)	0.69 (0.34, 1.40)
IV	62 (34)	0.63 (0.35, 1.12)	0.85 (0.44, 1.67)
HADS Depression (0–21)			
None (0–7)	114 (35)	1	1
Mild (8–10)	32 (32)	0.86 (0.54, 1.39)	0.92 (0.51, 1.64)
Moderate/severe (11–21)	16 (24)	0.59 (0.32, 1.08)	0.73 (0.32, 1.65)
HADS Anxiety (0–21)			
None (0–7)	95 (35)	1	1
Mild (8–10)	39 (34)	0.86 (0.56, 1.32)	1.20 (0.70, 2.05)
Moderate/severe (11–21)	27 (27)	0.69 (0.39, 1.22)	1.15 (0.61, 2.19)
Employment status			
Not employed	114 (34)	1	1
Employed	46 (33)	0.96 (0.63, 1.46)	0.79 (0.45, 1.40)
Lives alone			
No	139 (35)	1	1
Yes	25 (27)	0.76 (0.43, 1.16)	0.80 (0.44, 1.46)
Do you have someone to provide emotional support?			
Yes	142 (33)	1	1
No	4 (18)	0.45 (0.15, 1.35)	0.56 (0.14, 2.20)
No need	17 (43)	1.49 (0.77, 2.88)	1.11 (0.47, 2.65)
Do you have someone to rely on for extra help?			
Yes	132 (34)	1	1
No	8 (22)	0.53 (0.23, 1.19)	0.63 (0.21, 1.88)
No need	24 (34)	0.98 (0.57, 1.66)	0.66 (0.34, 1.28)

† Adjusted for all other listed variables

OR Odds ratio; 95%CI 95 percent confidence interval

HADS = Hospital Anxiety and Depression Scale

402 (82%) participants thought that prognostic information was important (31 did not, 59 'don't know', 10 missing) (Table 2). Again, this proportion was similar across all participating practices. This was highest among those currently in paid employment (adjusted OR 2.95; 95%CI 1.33, 6.57). All other associations were statistically non-significant. Perceived importance of prognostic information was strongly associated with recalled prognostic discussion in the consultation (crude OR 5.66; 95% CI 2.76, 11.59). Despite this, only 38.6% of those who thought that prognostic information was important recalled having this discussion with the GP in the consultation.

**Table 2:** Determinants of patient-perceived importance of prognostic information

	Prognostic information important N (%)	Crude OR (95% CI)	Adjusted† OR (95% CI)
Age (years)			
50–59	146 (84)	1	1
60–69	140 (85)	1.04 (0.57, 1.87)	1.92 (0.91, 4.07)
70–79	86 (80)	0.72 (0.39, 1.35)	0.98 (0.43, 2.25)
80+	30 (65)	0.35 (0.17, 0.72)	0.57 (0.20, 1.66)
Gender			
Female	243 (81)	1	1
Male	159 (84)	1.25 (0.77, 2.01)	1.07 (0.59, 1.95)
First consultation			
No	237 (80)	1	1
Yes	162 (85)	1.37 (0.84, 2.23)	1.62 (0.90, 2.90)
Self-rated general health			
Excellent/very good/good	272 (81)	1	1
Fair/poor	125 (84)	1.21 (0.72, 2.02)	1.12 (0.57, 2.18)
Chronic Pain Grade			
I	53 (82)	1	1
II	71 (73)	0.62 (0.29, 1.34)	0.70 (0.29, 1.68)
III	87 (81)	0.94 (0.43, 2.06)	1.11 (0.44, 2.76)
IV	157 (86)	1.42 (0.67, 3.03)	1.41 (0.56, 3.54)
HADS Depression (0–21)			
None (0–7)	257 (80)	1	1
Mild (8–10)	85 (85)	1.43 (0.78, 2.65)	1.89 (0.83, 4.29)
Moderate/severe (11–21)	56 (86)	1.57 (0.74, 3.35)	1.89 (0.62, 5.79)
HADS Anxiety (0–21)			
None (0–7)	217 (81)	1	1
Mild (8–10)	94 (81)	1.02 (0.59, 1.78)	0.87 (0.43, 1.75)
Moderate/severe (11–21)	85 (86)	1.46 (0.77, 2.76)	0.97 (0.41, 2.32)
Employment status			
Not employed	264 (78)	1	1
Employed	127 (90)	2.50 (1.36, 4.62)	2.95 (1.33, 6.57)
Lives alone			
No	333 (83)	1	1
Yes	69 (78)	0.71 (0.40, 1.24)	1.27 (0.60, 2.67)
Do you have someone to provide emotional support?			
Yes	350 (82)	1	1
No	16 (80)	0.88 (0.29, 2.71)	0.34 (0.08, 1.46)
No need	33 (83)	1.04 (0.44, 2.43)	1.45 (0.49, 4.29)
Do you have someone to rely on for extra help?			
Yes	320 (84)	1	1
No	30 (83)	0.97 (0.39, 2.43)	1.43 (0.35, 5.78)
No need	51 (72)	0.49 (0.29, 0.89)	0.36 (0.17, 0.78)

† Adjusted for all other listed variables

OR Odds ratio; 95%CI 95 percent confidence interval

HADS = Hospital Anxiety and Depression Scale

Several reasons were given as to why it was important to know what was likely to happen to their pain over the next six months. These included knowing for the sake of knowing, planning for the future (leisure, work, home and caring activities), knowing about pain in order to them help cope with it and allowing them to alter activity to prevent deterioration.

Reasons given for why it was not important to know what was likely to happen to the pain they consulted with included there being no point in knowing, that nothing could be done anyway, that progression was inevitable, that nothing could be done and that it couldn't be accurately predicted.

## Discussion

This is the first study to investigate how often prognostic discussion occurs during the consultation and how important patients feel prognostic discussion is for musculoskeletal pain. We found that while over 80% of older people with musculoskeletal pain feel prognostic discussion is important (and give clear reasons for this), prognosis may be discussed in only a third of consultations.

This study included a relatively large number of patients recruited from consecutive consulters presenting to several general practices, and representing a range of common musculoskeletal pain complaints. Although the response to the baseline questionnaire was high (77%) some groups, such as those with poor literacy skills, lower levels of formal education or non-English language speakers may be under-represented amongst responders, affecting the generalisability of our findings. Some information on the demographics of these groups is available. The population of East Cheshire is predominantly white British (93.9% for the whole of East Cheshire, compared with 84.2% white British for England as a whole). Furthermore, the percentage of participants describing their ethnic group as white in participating practices ranges from 97.5% – 98.7% and as such English language difficulties may be less of problem for this study than for similar research conducted elsewhere [20]. For this study we chose a local rather than a national sample frame. The participating practices are part of the Keele GP Research Partnership, which is supported by the North Staffordshire NHS Primary Care Research Consortium. Their registered populations are representative of the wide range of socio-demographic status found in the East Cheshire area.

The relatively low frequency of prognostic discussion in the consultation is based on patient recall and although the average time between consultation and receipt of the completed questionnaire was only 16 days, 40–80% of information in consultation is immediately forgotten by patients and recall may be poorer in older age groups [21]. It is possible that prognosis is discussed more often than our results would suggest. If so, our findings indicate that it is seldom memorable. Findings from an earlier survey of 68 GPs found that only 45% reported 'often or always' discussing the prognosis of musculoskeletal pain with older patients [22], which is consistent with our present findings. The use of other methodologies, such as audio or videotaping consultation could provide a more accurate picture of the extent of prognostic discussion during the musculoskeletal pain consultation. Patient understanding and interpretation of the prognostic questions presented in the questionnaire is also unclear. Whilst this cross-sectional analysis study has provided some insight into patient's views of the importance of prognostic discussion within the consultation, future qualitative research could provide more in-depth insights.

The proportion of older patients with musculoskeletal pain thinking prognostic information is important is similar to previous studies of other conditions including metastatic cancer, breast cancer, chronic obstructive pulmonary disease, congestive heart failure, dementia, and a range of physical symptoms [5-7,23] giving us further confidence our findings. Therefore the role of the clinician as "prognostician" probably ought to receive greater attention but our findings do not imply the need for a blanket policy of discussing prognosis with every patient in each consultation.

A minority of participants did not think that it was important to know what was likely to happen to their pain. The reasons for this are revealing. Some relate to common misconceptions that patients and professionals may have about musculoskeletal disorders ("it's just age", "nothing can be done to help it") [24]. A balance must be struck between challenging these misconceptions and recognising and respecting these patients' wishes. One approach would be to ask patients how much they want to know about the likely course of their condition [25], an approach that has been successfully described for other conditions.

We asked participants if they felt prognostic information on the likely course of their musculoskeletal pain was important. A majority of participants thought it was. Yet it cannot be assumed that all patients favourably disposed to receiving prognostic information would necessarily wish prognostic discussion to take place in their consultation or that such discussion would influence their health outcomes. These considerations are important and need to be evaluated before recommending that prognostic dialogue should become a routine part of the consultation for older people with musculoskeletal pain.

The value of prognostic information may be depend upon the specific nature, extent, and timing of such information. Our findings merely indicate the general beliefs in the importance of prognostic information and the apparent shortfall of information provision in practice. Further work is needed on which outcomes are of interest to patients (e.g. pain recovery, return to work, resolution of disability), how much information is most useful, and in what form prognostic information should be given. Although only a minority of patients in this study recalled discussing prognosis with their general practitioner, the problem may not be a lack of prognostic discussion in the consultation, but a lack of simple, useful, explicit information that patients understand and retain.

Giving greater priority to prognostic discussion in the time-constrained consultation must presumably be at the expense of something else. Our study did not ask patients to rate the importance of other aspects of the consultation (e.g. listening to their complaint, discussing diagnosis, considering treatment options) or rank the relative importance of prognostic information, although studies that considered this for other conditions have found that patients consistently rank prognostic information highly [26].

## Conclusion

In conclusion, our findings suggest that whilst most older patients with musculoskeletal pain felt information on prognosis is important, prognostic discussion within the consultation is only infrequently recalled. Researching the views of prognostic discussion with older patients with musculoskeletal pain is currently not as advanced as for other conditions such as cancer and COPD. The precise information needs of patients, the most suitable way to communicate this information, and the potential health consequences of such dialogue remain unknown, making the delivery of accurate and appropriate prognostic information a continuing challenge to healthcare professionals.

## Competing interests

The authors declare that they have no competing interests.

## Authors' contributions

CM and GP designed the study, were responsible for data collection, conduct and analysis. CM and GP wrote and revised the manuscript. Both authors read and approved the final manuscript.

## Pre-publication history

The pre-publication history for this paper can be accessed here:


